# Supplementary material for: Characterizing superspreading potential of infectious disease: Decomposition of individual transmissibility
Source: PLoS Comput Biol. 2022 Jun 27;18(6):e1010281. doi: 10.1371/journal.pcbi.1010281 (PMC9269899; doi:10.1371/journal.pcbi.1010281)
Supplement: Attachment — Submitted filename: superspreading.pdf [file pcbi.1010281.s001.pdf]

Re: Characterizing superspreading potential of infectious disease: Decomposition of individual transmissibility

To Whom It May Concern:

This paper attempts to extend the original statistical model formulated by *Llyod-Smith et al. 2005*, by replacing the negative binomial distribution by the so-called Delaporte distribution.

I have a few major comments:

(1) The language of the paper needs substantial improvement. The paper in the current form is difficult to read due to grammatical errors and poor choices of sentence structures throughout the paper. For example, in the Author Summary, this sentence is unnecessarily lengthy and difficult to comprehend: “The control strategy-making process may benefit from monitoring the transmission characterized under this decomposition framework, where for COVID-19 pandemic, population-wide interventions are suggested to contain the outbreak before recommending the high-risk-specific control strategies.” I also think the structure of paper can be significantly improved , e.g. why the section “Conclusion” is followed by “Results and discussion”?

(2) The authors claim that the main novelty of the paper is the decomposition of the offspring number (*X*) of each index case into two components: a fixed part (*X*F) and variable part (*X*V). However, it is not clear from the paper how we may interpret the additional parameters biologically or epidemiologically, compared to original model by Llyod-Smith et al. Is this purely a statistical exercise? The current paper fails to highlight the motivation of this approach.

Regards,
