# Supplementary material for: Characterizing superspreading potential of infectious disease: Decomposition of individual transmissibility
Source: PLoS Comput Biol. 2022 Jun 27;18(6):e1010281. doi: 10.1371/journal.pcbi.1010281 (PMC9269899; doi:10.1371/journal.pcbi.1010281)
Supplement: Attachment — Submitted filename: reponse_letter_R1.docx [file pcbi.1010281.s002.docx]

Dear Editor,

Thanks for your positive decision, and helpful comments on manuscript ID: **PCOMPBIOL-D-21-01784**, or EMID: **9e9c35502c254f31**. We have addressed all comments, and enriched the manuscript for your re-consideration.

Many thanks.

Sincerely,

Shi Zhao, and Daihai He (corresponding authors)

**Comments from the Editor**

**Comments:** Both Reviewers have raised excellent points that should be helpful to the authors to strengthen their work.

**Response:** Thanks for the positive decision. We appreciate the efforts in evaluating this submission, and we have revised the manuscript for your re-evaluation.

**Reviewer #1**

**Comments:** This paper attempts to extend the original statistical model formulated by Llyod-Smith et al. 2005, by replacing the negative binomial distribution by the so-called Delaporte distribution. I have a few major comments.

**Response:** Thanks for the comments. We appreciate the efforts in reviewing this submission, and we have revised the manuscript for your re-evaluation.

**Comments:** The language of the paper needs substantial improvement. The paper in the current form is difficult to read due to grammatical errors and poor choices of sentence structures throughout the paper. For example, in the Author Summary, this sentence is unnecessarily lengthy and difficult to comprehend: “The control strategy-making process may benefit from monitoring the transmission characterized under this decomposition framework, where for COVID-19 pandemic, population-wide interventions are suggested to contain the outbreak before recommending the high-risk-specific control strategies.” I also think the structure of paper can be significantly improved, e.g., why the section “Conclusion” is followed by “Results and discussion”?

**Response:** Thanks for the concerns. We have revised the manuscript to improve the readability (a professional editing service is used).

For the sentence pointed out by the reviewer, we have revised it as follows.

“*The disease control strategies may be informed by monitoring the characteristics of superspreading. For COVID-19 pandemic, population-wide interventions are suggested in the first place to limit the transmission, and then the high-risk-specific control strategies are further recommended to lower the superspreading risk.*”

For the structure of paper, we have merged the last paragraph into the “Results and discussion” section, and removed the “Conclusion” section.

**Comments:** The authors claim that the main novelty of the paper is the decomposition of the offspring number (X) of each index case into two components: a fixed part (XF) and variable part (XV). However, it is not clear from the paper how we may interpret the additional parameters biologically or epidemiologically, compared to original model by Llyod-Smith et al. Is this purely a statistical exercise? The current paper fails to highlight the motivation of this approach.

**Response:** Thanks for the concerns.

We consider both classic NB distribution, and the decomposition model (i.e., Delaporte distribution) in the manuscript are descriptive tools to capture the patterns in the stochasticity in disease transmission and variation in individual infectiousness. The goal is to seek for a ‘better model’ to describing the heterogeneity in transmission from the theoretical side as well as yield a satisfactory fitting performance from statistical side. As we have mentioned in the manuscript,

“*With respect to describing the heterogeneity in transmission from the theoretical side, candidate models are compared in their fitting performances to the real-world observations [29]. Inspired by the compounding relationship between Poisson and NB distributions, we consider that the composition of Poisson rate can be modelled with a more generalized framework.*”

The interpretation of the additional parameter, i.e., the fixed part of reproduction number R_F_, is linked to the classic NB framework, and also further generalized from a new angle. Unlike the individual reproduction number (λ) in the NB distribution, which is a Gamma-distributed variable. We further decomposed λ into a fixed part (*λ*_F_) and variable part (*λ*_V_), where *λ*_F_ = R_F_ is a constant (instead of variable). This means although λ is variable, there is an additive part (*λ*_F_) of λ is fixed, i.e., constant for each individual. As we have mentioned in the manuscript,

“*Delaporte distribution allows a decomposition of the individual reproduction number (λ) into two independent and additive components (i.e., λF and λV). Although the offspring cases (XF) generated from the λF part are variable, the fixed component λF = RF is a constant.*”

By contrast, for the variable part of λ, i.e., *λ*_V_, different insights on the dispersion parameter *k* also appears in comparing to the classic NB distribution. We have explained this side in the manuscript as follows.

“*As the fixed part (RF) of R vanishes in NB distribution, 1/√k is the coefficient of variation (CV) of the Gamma distribution that is followed by the individual reproduction numbers (λ). In the context of Delaporte distribution, the effect of k in shaping the variation of λ is restricted to the CV of its variable part (λV), which is also Gamma-distributed.*”

We also remark that another novelty of the decomposition model (i.e., Delaporte distribution) is to extend to other commonly used measurement of superspreading such as one-generation cluster size, and final outbreak size, as we have elaborated in section 2.5 in details, which are very useful to quantify the superspreading potential by using real-world observations.

**Reviewer #2**

**Comments:** This paper provides an interesting viewpoint on the potential use of Delaporte distribution for the offspring distribution as an extended alternative to the negative-binomial distribution. The explanation of the methods is clear and well supported. Inclusion of not only offspring distribution data but also single-generation and final cluster size data is very useful. Moreover, some of the study findings, including that the temporal change in South Korean data is explained by the change in the R_F/R_V proportion (rho), not in k, are of epidemiological interest. This may bring a new insight into how we may understand the offspring distribution from outbreaks under interventions.

Overall, I feel the manuscript is of good quality and do not have much major criticism. Meanwhile, I would like to list two suggestions which I believe could further improve the epidemiological relevance of this paper; if the authors believe these do not make enough sense or involve overwhelming amount of additional effort (which is not what I wish to impose), I would be happy to be convinced.

**Response:** Thanks for the positive comments. We appreciate the efforts in reviewing this submission, and we have revised the manuscript for your re-evaluation.

**Comments:** While some may adopt the Delaporte distribution for characterizing the offspring distribution, I expect that the majority of studies might continue to use the negative-binomial distribution (because it’s more mathematically convenient). In that case, it might be important to ensure that the interpretation of the estimates from these distributions are reasonably linked. Specifically, a study may fit a negative binomial distribution to a dataset (let’s call it dataset A) which is actually better represented by a Delaporte distribution, and report estimates for negative-binomial R and k. If one wishes to analyse another dataset (dataset B) using a Delaporte distribution and compare the results with these estimates, it would be convenient to have a measure that quantifies the similarity between a negative-binomial and a Delaporte distribution. For example, the estimated distributions in Figure 5 are more or less very similar between the NB and Delaporte, however their estimates are very different (because they represent different quantities). If the Delaporte distribution is close enough to a negative binomial distribution, NB might be a practical choice to ensure comparability and/or mathematical convenience (even if AIC suggests Delaporte is preferred). It would be therefore nice to have the following statistics given the Delaporte parameters R_F, R_V, and k: (1) estimators for R and k of a negative distribution that is the most similar to the given Delaporte distribution; (2) a measure of the similarity between the Delarporte distribution and the negative distribution given by (1), or conditions for the NB approximation to work well. However, these may not be easily obtained from the Delaporte estimates (e.g., require nonlinear optimization), in which case I would not request it as strongly.

**Response:** Thanks for the suggestion.

We agree that linking a Delaporte distribution to a NB distribution would be of practically interest. For simplicity, we would propose to approximate the R and k of NB distribution by using the moments of a given Delaporte distribution. We have added the following contents in the manuscript for elaboration.

“*In practical analysis, one may also be interested in obtaining estimators for R and k given the parameter estimates of Delaporte distribution. Although the closest theoretical form may be complex to derive, convenient approximation by using moments of the Delaporte distribution could be considered as a rapid approach. To distinguish the dispersion parameters, we denote k_NB_ and k_D_ for NB and Delaporte distributions, respectively. For a given Delaporte distribution, the first moment (i.e., mean) is R_F_ + R_V_, and the second central moment (i.e., variance) is* $R_{F}+R_{V}\left( 1+\frac{R_{V}}{k_{D}} \right)$*. Thus, if let the NB distribution have the same value of mean and variance, for the approximated NB distribution, we have* $\hat{R}=R_{F}+R_{V}$*, and* $\hat{k_{NB}}=\left( \frac{\hat{R}}{R_{V}} \right)^{2}k_{D}=\left( \frac{R_{F}+R_{V}}{R_{V}} \right)^{2}k_{D}=\frac{k_{D}}{\left( 1-\rho\right)^{2}}$*. Although this approximation can be directly calculated rapidly, by using the estimates of the example offspring datasets, we remark that* $\hat{k_{NB}}$ *here appears slightly low than the posterior estimates of* $k_{NB}$ *in Table 1.*”

For the “*measure of the similarity between the Delarporte distribution and approximated NB distribution*”, we consider the standard way is (still) relying on the likelihood theory as we have discussed using likelihood ratio test or (almost equivalently) AIC-based model selection. It is very difficult to define similarity without computational practice involving the sample data and theoretical models. Even we could define a “*similarity*” metric between two theorical probability distributions (without considering the data used to infer the parameters), e.g., by measuring distance of CDF or PDF etc, the approach may lose the initial intuition of this study, which is to find a decent descriptive model capturing the heterogeneity in individual infectiousness with real-world observations. As such, it seems less practically meaningful to do so in this manuscript. Of course, we are happy to correct ourself if wrong.

**Comments:** If there is an appropriate dataset available, I would wish to see an application example of the Delaporte distribution to the final outbreak size c, in addition to the currently included offspring distribution data (e.g., what correspond to Fig 5 and Table 1).

**Response:** Thanks for the suggestion. We have adopted the final outbreak size dataset of MERS reported in Eurosurveillance 2014;19(23):20824, which was also studied in Eurosurveillance 2015;20(25):21167. We estimated the model parameters by using Delaporte distribution and also NB distribution, and further compared by AIC. We found weak evidence that Delaporte distribution may (slightly) outperform NB distribution in fitting this dataset. We have included the following content in the main text for elaboration.

“*To illustrate the translation from final outbreak size probability in Eqn (7) to likelihood-based estimation, we adopted the final outbreak size observations of Middle East respiratory syndrome coronavirus (MERS-CoV) infections in the Middle East region, which was reported in [87]. The dataset has a sample size of 55 including a total of 104 laboratory-confirmed MERS cases, and all final outbreaks were seeded by single cases, which was also summarized and studied in [3]. As such, Eqn (8) is employed to construct the likelihood function for Delaporte distribution. We estimated RF at 0.17 (95%CrI: 0.03, 0.45), RV at 0.32 (95%CrI: 0.01, 1.53), and k at 0.04 (95%CrI: 0.00, 0.19) with AIC of 114.60. We also repeated the estimation by using the NB distribution, which leads to R at 0.47 (95%CrI: 0.30, 0.78) and k at 0.27 (95%CrI: 0.10, 0.98) with AIC of 115.68. For the previous estimates using NB in [3], it was estimated R at 0.47 (95%CrI: 0.29, 0.80) and k at 0.26 (95%CrI: 0.09, 1.24), which was in line with our estimates. The k estimate appears lower in Delaporte distribution, and the ρ estimate at 0.33 (95%CrI: 0.05, 0.98) was larger than 0 such that the fixed part of R was evident, which was also indicated by the difference in AIC values.*”

Minor comments

**Comments:** L122 addictive -> additive?

**Response:** Revised with thanks.

**Comments:** L124 I believe the individual reproduction number here is rather a non-standard usage (e.g. see https:doi.org/10.1371/journal.pone.0000758) and thus I feel this needs to be redefined/explained.

**Response:** Thanks for the concern. Our definition on the ‘individual reproduction number’ follows the definition in Llyod-Smith et al. Nature. 2005;438(7066):355-9. We were also aware the classic paper mentioned by the reviewer a long time ago. By comparing the definitions in these two papers, we consider they are consistent because

- both definitions treat the individual reproduction number as a random variable linking to a probability distribution defined by the reproduction number at population scale, and
- both definitions concentrate on a particular given seed case (instead of in the context of general population).

As such, we have revised this part as follows for clarification, and also cited these two papers in the main text.

“*Referring to the definition in (29), λ conceptualized the individual reproduction number (51), which is a random variable and represents the expected number of secondary cases caused by a (particularly) given primary case.*”

If we misunderstand the concept, we are happy to correct.

**Comments:** L163–4 can you add a bit more context such that it’s clear in what way the interpretation of k is consistent with NB?

**Response:** Thanks for the suggestion. We consider the interpretation of k is generalized in the context of Delaporte distribution in comparing to NB distribution. Thus, we have added to following contents for elaboration.

“*The implementation of Delaporte distribution is considered as a generalization of the framework proposed in [29], and thus the interpretation of dispersion parameter k generalizes it meaning in NB distribution [45]. As the fixed part (R_F_) of R vanishes in NB distribution, 1/√k is the coefficient of variation (CV) of the Gamma distribution that is followed by the individual reproduction numbers (λ). In the context of Delaporte distribution, the effect of k in shaping the variation of λ is restricted to the CV of its variable part (λ_V_), which is also Gamma-distributed.*”

**Comments:** L176 I believe ⌊∙⌋ is the floor function, not rounding (and the floor function should be used here).

**Response:** Thanks for pointing out, and sorry, it was a typo. We have corrected as “*⌊∙⌋ denotes the floor function, which outputs the largest integer less than or equal to the given number*”.

**Comments:** Section 2.4: In this setting I believe AIC and LR test are equivalent (difference of 2 in AIC corresponds to p = 0.05 in LR test) and including both seems redundant (and AIC is more widely used because it’s a more general framework). Could the same conclusion be obtained if the AIC-based model selection was used in place of LR for the discussion of type I/II errors?

**Response:** Thanks. We agree that the AIC-based criterion for model selection here is equivalent to LR test, and yes, as pointed out by reviewer, “*the same conclusion can be obtained if the AIC-based model selection was used in place of LR for the discussion of type I/II errors*”. The reason we adopt LR test for evaluating type I/II errors is to calculate statistical significance (i.e., p-value), which is more straightforward for clinical researcher. For clarification, we have added the following contents in the main text.

“*Note that statistically, the p-value < 0.05 from LR test here is (roughly) equivalent to the AIC-based model selection with a cutoff of 2 units.*”

**Comments:** L315 It -> It is?

**Response:** Revised with thanks.

**Comments:** L354–357 This is an important point. Can you briefly mention where the assumption of R<1 was introduced (L334) that this assumption would be relaxed in the end? Otherwise, the reader may misinterpret that the final cluster size likelihood can only be used for R<1.

**Response:** Thanks for the concerns. We confirm that although final outbreak size observations are frequently used in the context of R<1, they could also be used for scenarios with R>1 (with adjustment on the likelihood framework). Thus, we have added the following content in at the ‘R<1’ part for clarification.

“*Although the final outbreak size is frequently linked to subcritical transmission, the final outbreak size may also be observable for supercritical transmission (R > 1), which we will introduce below more rigorously.*”

In the technical part (of the same section), we have also included the following content for elaboration.

“*… the formula in Eqn (7) was also applicable for supercritical transmission. When R > 1, there is a chance of* $\left[ 1-\sum_{c=i}^{\infty} \omega_{D}(c,i) \right]$ *that the outbreak will never extinct, which means the final outbreak size c becomes a defective random variable. Based on the property of branching process, we may calculate the probability of outbreak extinction* $\varepsilon$ *by solving* $\varepsilon=\left[ g_{D}\left( \varepsilon\right) \right]^{i}$*. Thus, the likelihood function can also be constructed by adjusting* $\varepsilon$ *as the denominator for supercritical transmission, which could be useful for final outbreak size observations while the epidemic is still ongoing.*”

**Comments:** Figures: there are an overwhelming number of figures (with even many panels). Some of them may better fit in the supplementary file.

**Response:** Thanks for the concern. Since the journal ‘PLoS Computational Biology” encourages to “*provide details of all of the experiments that are required to support the conclusions of the paper*” (link: <https://journals.plos.org/ploscompbiol/s/submission-guidelines#loc-parts-of-a-submission>) in the results section. Hence, we would prefer to maintain the current figure contents. For the multi-panel issue, as it is, most of panels are tabulated in one figure for comparison among each other to show the trend changes.

**Comments:** Throughout, I suggest the authors always distinguish between one-generation cluster size and final outbreak size and try to avoid just saying “cluster size” or “outbreak size” for best clarity.

**Response:** Thanks for the suggestion, and we agree. We have check throughout the manuscript to ensure consistency and clarity.

**Comments:** I found some typos/grammatical errors in the manuscript (which I named above) and I suggest the authors proofread the manuscript once again.

**Response:** Thanks for the concerns. We have double checked the language in the manuscript to correct typos and errors for re-evaluation.
